# Whom and Where Are We Not Vaccinating? Coverage after the Introduction of a New Conjugate Vaccine against Group A Meningococcus in Niger in 2010

**DOI:** 10.1371/journal.pone.0029116

**Published:** 2012-01-20

**Authors:** Sung Hye Kim, Lorenzo Pezzoli, Harouna Yacouba, Tiekoura Coulibaly, Mamoudou H. Djingarey, William A. Perea, Thomas F. Wierzba

**Affiliations:** 1 Translational Research Division, International Vaccine Institute, Seoul, Republic of Korea; 2 Consultant for the Meningitis Vaccine Project, London, United Kingdom; 3 Department of Surveillance, Statistics and Epidemic Response, Ministry of Public Health, Niamey, Niger; 4 Expanded Programme on Immunization, World Health Organization, Niamey, Niger; 5 Meningitis Vaccine Project Focal Point, Inter-country Support Team for West Africa, World Health Organization, Ouagadougou, Burkina Faso; 6 Epidemic Readiness and Intervention, World Health Organization, Geneva, Switzerland; Health Protection Agency, United Kingdom

## Abstract

MenAfriVac is a new conjugate vaccine against *Neisseria meningitidis* serogroup A developed for the African “meningitis belt”. In Niger, the first two phases of the MenAfriVac introduction campaign were conducted targeting 3,135,942 individuals aged 1 to 29 years in the regions of Tillabéri, Niamey, and Dosso, in September and December 2010. We evaluated the campaign and determined which sub-populations or areas had low levels of vaccination coverage in the regions of Tillabéri and Niamey. After Phase I, conducted in the Filingué district, we estimated coverage using a 30×15 cluster-sampling survey and nested lot quality assurance (LQA) analysis in the clustered samples to identify which subpopulations (defined by age 1–14/15–29 and sex) had unacceptable vaccination coverage (<70%). After Phase II, we used Clustered Lot Quality Assurance Sampling (CLQAS) to assess if any of eight districts in Niamey and Tillabéri had unacceptable vaccination coverage (<75%) and estimated overall coverage. Estimated vaccination coverage was 77.4% (95%CI: 84.6–70.2) as documented by vaccination cards and 85.5% (95% CI: 79.7–91.2) considering verbal history of vaccination for Phase I; 81.5% (95%CI: 86.1–77.0) by card and 93.4% (95% CI: 91.0–95.9) by verbal history for Phase II. Based on vaccination cards, in Filingué, we identified both the male and female adult (age 15–29) subpopulations as not reaching 70% coverage; and we identified three (one in Tillabéri and two in Niamey) out of eight districts as not reaching 75% coverage confirmed by card. Combined use of LQA and cluster sampling was useful to estimate vaccination coverage and to identify pockets with unacceptable levels of coverage (adult population and three districts). Although overall vaccination coverage was satisfactory, we recommend continuing vaccination in the areas or sub-populations with low coverage and reinforcing the social mobilization of the adult population.

## Introduction

The African “meningitis belt” is an area that stretches from Senegal to Ethiopia, where major epidemics of meningococcal meningitis regularly occur [1]. *Neisseria meningitidis* serogroup A is the primary cause of meningitis epidemics in the meningitis belt [2]. The human toll from these epidemics is enormous. In the 1996–1997 epidemics, more than 250,000 cases and 25,000 deaths were reported [3]. During the 2009 epidemic season, 14 countries reported a total of 78,416 cases, including 4,055 deaths. After Nigeria, Niger was the second most affected country [2].

The Meningitis Vaccine Project (MVP) was created by the World Health Organization (WHO) and the Program for Appropriate Technology in Health (PATH) with the goal of eliminating meningococcal epidemics in Africa through the development, licensure, introduction, and widespread use of conjugate meningococcal vaccines [4]. Thanks to a grant from the Bill & Melinda Gates Foundation, an affordable (less than US$ 0.50 per dose), effective, and long-lasting meningococcal A conjugate vaccine (MenAfriVac) was developed by the MVP and manufactured by the Serum Institute of India [5]. MenAfriVac was pre-qualified by WHO in June 2010 [6] and introduced in multiple phases in Burkina Faso, Mali and Niger, to optimize the preparation for its widespread use in Africa [6], [7]. During the first phase (Phase I) of the introduction campaigns, pregnant or lactating women were not recommended to receive the vaccine due to limited data on vaccine safety in these populations [7]. This recommendation was revised for the following phases since the Global Advisory Committee on Vaccine Safety (GACVS) considered that benefit of meningitis vaccination outweighed the theoretical risk of adverse events following immunization (AEFI) for pregnant or breastfeeding women (WHO Technical Note 22/11/2010: MenAfriVac vaccine campaigns in the African meningitis belt: use of vaccine in pregnant and lactating women).

In Niger, the Phase I campaign was conducted in the district of Filingué in the region of Tillabéri in September 2010. The second phase of the introduction (Phase II) was conducted in the regions of Niamey, Tillabéri, and Dosso in December 2010.

The WHO Expanded Programme on Immunization (EPI) recommends two methods for the evaluation of vaccination campaigns: cluster sampling to obtain estimates of vaccination coverage in a defined area [8] and lot quality assurance sampling (LQAS) to identify possible pockets of low coverage [9], [10]. After each of the two phases, we combined cluster sampling and LQAS to evaluate vaccination coverage and to determine whether some sub-populations or districts had unacceptably low levels of coverage in order to assist future vaccination strategies.

## Methods

### Ethics Statement

The study protocol was ratified by the National Campaign Organization Committee chaired by the Ministry of Health in Niger. Ethical approval for the survey was granted locally by the Ministry of Health of Niger as part of the standard procedure to evaluate national immunization campaigns. The International Vaccine Institute's Institutional Review Board also reviewed and waived the need for approval.

### Phased Vaccination Campaigns

The campaigns targeted individuals between 1 and 29 years of age, representing approximately 70% of the total population. The Ministry of Health organized the introduction campaigns adopting fixed, outreach, and mobile strategies according to local characteristics of the vaccinating areas. Each vaccinated individual received a vaccination card and each vaccine dose was recorded in a tally sheet to count how many doses were administered by age group and per day. In addition, social mobilization activities about the benefit of this new vaccine were undertaken before and during the campaigns.

Phase I was conducted in the district of Filingué in the region of Tillabéri from 21 to 27 September with the objective of vaccinating at least 90% of the target population. After Phase I, administrative coverage was 85%, thus the decision was made to conduct mop-up activities from 7 to 9 October 2010. Phase II was conducted in the regions of Niamey (three districts predominantly urban), Tillabéri (five districts predominantly rural), and Dosso (two districts predominantly rural) from 7 to 16 December 2010 with the same objective.

### Cluster Sampling Survey with Nested Lot Quality Assurance Analysis in Four Sub-Populations after Phase I

After Phase I, we conducted a cluster sampling survey in the target district of Filingué between 24 October and 2 November 2010. The study population was represented by the 392,221 individuals aged 1–29 years reported living in Filingué (National Population Census 2001, Niger Institute of National Statistics). We calculated a sample size of 450 subjects divided into 30 clusters of 15 (confidence interval: 95%; precision: 5%; expected coverage: 85%; design effect: 2) [11].

We nested the lot quality assurance (LQA) methodology in the cluster sample to assess if each of four sub-populations, defined as lots (Lot 1: boys aged 1–14 years; Lot 2: girls aged 1–14 years; Lot 3: men aged 15–29 years; Lot 4: women aged 15–29) had reached a minimum acceptable level of 70% vaccination coverage. In accordance with LQA methods [12], [13], we set the upper coverage threshold (UT) to 90% and the lower coverage threshold (LT) to 70%, yielding a decision value (d) of 5 in a sample (N) of 30, with alpha (the probability of classifying a lot as acceptable with unacceptable coverage) at 8% and beta (the probability of classifying a lot with acceptable coverage as unacceptable) at 7%. Operationally, this meant that if 5 or less unvaccinated individuals were found in the sample of 30, then the lot would be classified as acceptable (i.e. coverage is equal or above 70%); if 6 or more unvaccinated were found then it would be classified as unacceptable.

In each of the 30 clusters, we considered the first individual sampled per lot for the nested LQA analysis. In case not all lots were represented in the cluster sample of 15 individuals, surveyors were instructed to continue selecting individuals until all four lots were represented. If additional samples were needed for the LQA analysis, they would not be included in the estimation of vaccination coverage by cluster sampling, since the probability of selection would have been different.

### Clustered Lot Quality Assurance Sampling Survey after Phase II

After Phase II, we conducted a series of clustered lot quality assurance sampling (CLQAS) surveys at district level in the regions of Niamey (Districts: I, II, and III) and Tillabéri (Districts: Kollo, Say, Ouallam, Téra, and Tillabéri) between 19 and 28 December 2010. The study population was represented by the 2,136,287 individuals aged 1 to 29 years, residing in the two regions (excluding the district of Filingué already covered in Phase I).

According to the CLQAS methodology, we defined each district as a lot and designed different sampling plans with N = 100 divided in 10 clusters (k) of 10 individuals (n) and calculated alpha and beta based on different coverage thresholds assuming that coverage in the clusters (n = 10) would not vary more than 0.1 standard deviations from the coverage in the lot (N = 100) [14]. We eventually choose the plan with UT = 90%, LT = 75%, and d = 16 since it provided the lowest error levels: alpha≤4% and beta≤8%. The decision rule was as follows: if up to 16 unvaccinated individuals were found in each lot (district), then the lot was classified as acceptable with at least 75% vaccination coverage; if 17 or more unvaccinated were found then it was classified as unacceptable without achieving 75% of coverage.

We aggregated the data from the lots by adjusting them for the population size and for the size of the household (total number of target individuals living in the selected household) to estimate coverage in each of the two regions (excluding Filingué). In the region of Niamey, the sample of 300 individuals (3×10×10) would be sufficient to achieve an accuracy of 5% with a 95% confidence interval (CI), if we assumed 90% vaccination coverage and deff = 2. In the region of Tillabéri, the sample of 500 (5×10×10) would be sufficient to achieve an accuracy of 5% with a 95% CI, if we assumed 80%vaccination coverage and deff = 2.

### Sampling Procedure

The clusters (villages) were selected in each district using probability proportionate to population size (PPS) as described in two-stage cluster sampling methods [11] . We selected the first household in each cluster according to geographic random sampling: we drafted a map of the locality, divided it into smaller sectors according to existing divisions (streets, rivers, etc), and selected one sector according to simple random sampling (SRS).

In each household, we listed all the residents aged 1–29 and selected one with SRS for the interview. If the selected individual was not reachable, we moved to the subsequent household. If the selected participant was under 12 years of age, an older member of the household was invited to answer the questionnaire for that child. If only children under 12 years were present in the house, we did not conduct the interview and moved to the subsequent household instead.

### Outcome Measures and Definitions

We defined “vaccinated” as a person aged between 1–29 years who had received the MenAfriVac during the campaigns.

We measured vaccination coverage on two levels: based on the exclusive availability of the hand-held vaccination card (card only) and considering also if the individuals (or their care-takers) were verbally reporting as having been vaccinated (card+history). During the Phase I evaluation we collected information on the awareness about the campaign and on reasons for non-vaccination using multiple choice questions. During the Phase II evaluation we additionally collected information on the awareness about the hypothesized ten years length of protection of the vaccine. We used a structured pre-tested questionnaire for data collection.

### Statistical Analysis

Data entry and cleaning were conducted in Excel 2007 (Microsoft Corporation).

Analyses were conducted with STATA version 11 (STATA Corp). We used the “svyset” command for complex survey designs, using the population size of the districts and the number of eligible individuals living in the households to define the survey weights in order to account for differences in probability of selection. We obtained coverage estimates with 95% CI using the “svy:proportion” command.

We compared survey results with reported administrative coverage (the number of vaccine doses administered divided by the number of persons in the target population) and the coverage estimates from the region of Niamey (a predominantly urban region) with the estimates from Tillabéri (predominantly rural).

We combined the results from Phase I and Phase II to obtain an overall estimation of MenAfriVac vaccination coverage for the regions of Niamey and Tillabéri, using the formula for aggregating the results of multiple surveys conducted in different strata [8], [15].

## Results

### Phase I

We conducted 450 interviews in Filingué. Female respondents were 230 (51.1%). Estimated vaccination coverage was 77.4 (95% CI 70.2–84.6) by card only and 85.5% (95% CI: 79.7–91.2) by card or verbal history (Table 1). The majority (90.5%) of the 380 individuals who claimed to be vaccinated showed the vaccination card during the interview. Reasons for non-vaccination are presented in Table 2.

**Table 1:** MenAfriVac vaccination coverage in persons aged 1–29 years for the two phases presented by administrative coverage and coverage survey results, Niger, October–December 2010.

			Survey				
				Card Only		Card+History	
Phase	Population	Administrative Coverage	Sampled	Vaccinated	Estimated Coverage [95%CI]	Vaccinated	Estimated Coverage [95%CI]
I	Total	90.7%	450	340	77.4% [70.2–84.6]	380	85.5% [79.7–91.2]
II	Total	103.6%	800	647	81.5% [77.0–86.1]	747	93.4% [91.0–95.9]
	Niamey	107.1%	300	221	75.7% [65.9–85.4]	276	92.7% [88.5–96.8]
	Tillabéri	102.0%	500	426	84.3% [79.3–89.3]	471	93.8% [90.7–96.9]
	1–14	101.9%	563	465	83.4% [78.8–88.0]	539	96.0% [94.0–98.1]
	15–29	107.0%	237	182	77.0% [69.5–84.5]	208	87.2% [81.3–93.0]
	Male	-	328	266	83.2% [77.8–88.6]	311	95.8% [92.9–98.7]
	Female	-	472	381	80.4% [74.8–86.0]	436	91.8% [88.7–94.9]

**Table 2:** Reasons for non-vaccination in the individuals reportedly not vaccinated during Phase I (n = 70) and Phase II (n = 53) of the MenAfriVac introduction campaign, Niamey and Tillabéri, Niger, September–December 2010.

	Phase I	Phase II
Reason for non-vaccination	Result (%)	Result (%)
Being pregnant or breastfeeding	21 (30.0)	0 (0)
Absent during the time of vaccination	20 (28.6)	13 (24.5)
Vaccinator was absent when they presented for vaccination	16 (22.9)	8 (15.1)
Lack of information about the campaign	4 (5.7)	7 (9.4)
Sick during the time of vaccination	4 (5.7)	8 (15.1)
Vaccine was out of supply	0 (0)	3 (5.7)
Refusal/Not interested in vaccination	3 (4.3)	9 (17.0)
No more cards available	1 (1.4)	0 (0)
Vaccination site too far	0 (0)	1 (1.9)
Time of vaccination not convenient	1 (1.4)	1 (1.9)
Other	0 (0)	3 (5.7)
Total	70 (100)	53 (100)

The nested LQA analysis was completed in the four lots using the cluster survey data. The sample of 450 was sufficient to obtain the 120 sampling units (30×4) without recruitment of additional individuals. Based on the presence of the vaccination card, we classified the two sub-populations of 1–14 years of age as acceptable with vaccination coverage above 70% and the two of 15–29 years as unacceptable (Table 3).

**Table 3:** Vaccination coverage results in persons aged 1–29 years for Phase I of the first phase of the MenAfriVac introduction campaign presented by administrative coverage and Lot Quality Assurance (LQA) analysis, Filingue, Niger, October 2010.

		LQA Analysis (N = 30)				
			Card Only		Card + History	
Age Group	Administrative Coverage	Lot	Unvaccinated	Decision	Unvaccinated	Decision
1–14 years	98.6%	Males 1–14 years	5	Accepted	3	Accepted
		Females 1–14 years	4	Accepted	2	Accepted
15–29 years	74.0%	Males 15–29 years	10	Rejected	6	Rejected
		Females 15–29 years	18	Rejected	16	Rejected

### Phase II

Between 19 and 28 December 2010, we conducted 800 interviews in the regions of Niamey and Tillabéri (excluding Filingué). The majority (472; 59.2%) of respondents were female. Estimated vaccination coverage was 81.5% (95%CI: 77.0–86.1) by card only and 93.4% (95% CI: 91.0–95.9) by card or verbal history (Table 1). The majority (87.3%) of the 747 vaccinated individuals showed the vaccination card during the interview. Two individuals reported being vaccinated elsewhere (one in Burkina Faso and one in Filingué) but did not have the vaccination card; they were considered vaccinated as confirmed by verbal history in the analysis.

Seventy-eight percent (95%CI: 71.2–84.7) of respondents were aware that the campaign was conducted with a new vaccine against meningitis and 70.5% (95%CI: 63.0–78.0) of them were aware that its duration of protection is assumed to be up to ten years. Reasons for non-vaccination are presented in Table 2.

The CLQAS surveys were completed in the eight districts (lots). Based on card-confirmed vaccination status, five districts were classified as acceptable with vaccination coverage equal to or above 75%; while three were classified as unacceptable with coverage below 75% despite presenting administrative coverage above 100%. Allowing also for verbal confirmation, all districts were accepted (Table 4).

**Table 4:** Vaccination coverage results in persons aged 1–29 years for the second phase of the MenAfriVac introduction by administrative methods and Clustered Lot Quality Assurance Sampling (CLQAS) survey, Niamey and Tillabéri, Niger, December 2010.

			CLQAS Survey (N = 100)			
			Card Only		Card + History	
Region	District/Lots	Administrative Coverage	Unvaccinated	Decision	Unvaccinated	Decision
Niamey	Niamey I	107.4%	16	Accepted	9	Accepted
	Niamey II	106.3%	18	Rejected	5	Accepted
	Niamey III	108.5%	19	Rejected	10	Accepted
Tillabéri	Tillabéri	98.3%	6	Accepted	4	Accepted
	Ouallam	100.6%	6	Accepted	5	Accepted
	Kollo	97.9%	12	Accepted	8	Accepted
	Say	112.8%	17	Rejected	9	Accepted
	Téra	102.2%	4	Accepted	3	Accepted

### Overall Vaccination Coverage for Phases I and II

After the two phases, the overall estimated vaccination coverage against meningococcal A in Tillabéri and Niamey was 80.9% (95% CI: 74.4–87.4) documented by card only and 92.2% (95% CI: 87.0–97.4) considering card or verbal history of vaccination; 87.6% (987/1127) of the people considered vaccinated showed the vaccination card.

## Discussion

We evaluated the first two phases of the MenAfriVac introduction campaign in the regions of Tillabéri and Niamey in Niger using a combination of cluster-sampling and LQAS techniques. Overall vaccination coverage was 81% based on the availability of vaccination cards; 92% considering also verbal report of vaccination as a reliable source of information. After Phase I, we identified two sub-populations with unacceptably low coverage (<70%): males and females aged between 15–29 years considering both vaccination cards only and verbal history of vaccination. After Phase II, we identified three districts out of eight with unacceptably low vaccination coverage (<75%) because of the absence of vaccination cards; relying on verbal confirmation of vaccination status, we classified all three as with acceptable coverage. If this new conjugate vaccine against Meningococcus A is as effective in preventing meningococcus carriage as the Meningococcal C conjugate vaccine, the level of coverage achieved would be sufficient to provide herd immunity in the population, even with the most conservative assumption that 74.4% (lower confidence limit for the overall coverage estimate based on card retention) of people aged 1–29 (70% of the general population) were vaccinated, [16]–[19].

The fact that 88% of individuals who claimed to be vaccinated were able to show the card suggests that the population understood its importance. However, towards the end of the Phase II campaign, some districts reported shortage of vaccination cards, which could explain why we rejected three districts based on card confirmed vaccination status but accepted them considering verbal vaccination status.

After Phase I, the most common reason for non-vaccination was being pregnant or breastfeeding. This is not surprising since, at that time, pregnant or lactating women were not recommended to receive the vaccine. Being absent was the second most common reason for Phase I and the first for Phase II. This finding was expected for Phase I since the campaign was conducted only in one district in a shorter period of time, so individuals who were commuting to or working in outer areas of the district would have not been able to receive the vaccine; it is more surprising for Phase II since the campaign covered a much larger area than Phase I and was accompanied by stronger social mobilization activities. Sixteen unvaccinated individuals for Phase I and eight for Phase II reported not being vaccinated because the vaccinators were absent from their post, suggesting that there may have been programmatic issues in organizing vaccination activities, especially for Phase I. Overall twelve unvaccinated individuals refused the vaccine, while eleven were not vaccinated as a result of insufficient information. These results suggest communication to the population should be reinforced.

The proportion of individuals who were aware that the campaign was conducted with a new vaccine against meningitis was much higher in Phase II than in Phase I, suggesting that the social mobilization may have been more effective in Phase II in terms of delivering messages regarding the vaccine.

Administrative coverage can be used to take operational decisions following vaccination campaigns [20]–[22]. Levels of the administrative coverage ranged from 91% to 113% in the nine districts under evaluation. Coverage estimates were well below post-campaign administrative coverage. This discrepancy can be explained by two reasons: firstly, the denominator used to calculate the administrative coverage may be out of date or underestimated; secondly, it is possible that vaccines were administered to individuals who are out of the target group (1–29 years of age) or not included in the denominator (e.g. new residents, migrant workers, nomadic population, etc.).

The cluster sampling method provides a coverage estimate with 95% CI for the entire territory or population under study, but it does not provide information on pockets of unvaccinated individuals [23]. Depending on levels of expected coverage, cluster sampling surveys would require sample sizes in the order of hundreds individuals to obtain reliable estimates [11]. After Phase I, to identify unvaccinated pockets, we decided not to conduct cluster sampling surveys in sub-populations. Instead, we nested the LQAS methodology in the cluster sampling survey and identified that both the female and male adult groups had low vaccination coverage. With regards to the female adult group, this was not surprising, since pregnant or breastfeeding women were not recommended to receive the vaccine during Phase I. As for adult males, this population has not been usually the target of vaccination campaigns, and may have not been aware of the fact that the campaign was targeting them too.

After Phase II, we wanted to verify whether each targeted district had achieved at least 75% of coverage. We also intended to test if coverage in Tillabéri, which reported 102% of administrative coverage, was lower than coverage in the region of Niamey, which was reported as 107%. We applied the CLQAS methodology and obtained results at multiple levels. We confirmed that three districts (Niamey II, III, and Say), all with administrative coverage levels above 100%, had not achieved 75% coverage by card retention, showing that making operational decisions only relying on administrative coverage has a high chance of missing vulnerable populations in need. Second, we obtained coverage estimates at regional and higher level, showing that the coverage estimates (especially by verbal history) were not significantly different between Tillabéri and Niamey. Niamey, the capital city, is encircled by Tillabéri and has more residents or commuters than registered, which is vice versa for Tillabéri. Our result suggests that administrative coverage might have been overestimated, especially in the region of Niamey, where many individuals were not included in the denominator even if they were actually vaccinated.

Nesting the LQA analysis in the cluster survey or aggregating data from CLQAS allowed us to conduct the survey with greater feasibility and to obtain more information than we would have normally obtained from traditional cluster surveys. One previous study has explored the applications of nesting LQAS in cluster sampling to compare power and precision of different study designs to assess the prevalence of acute malnutrition [24]. In our case, we nested LQA in the cluster sample to assess vaccination coverage at sub-population level rather than to compare the two methods. CLQAS demonstrated to be an efficient methodology in the context of Phase II, which covered vast areas of the country. As expected, clustering sampling points in lots increased the feasibility of the survey in the field [15], [25], since the teams had to travel only to ten locations per district.

This study was subject to a number of limitations. Firstly, since we have instructed surveyors not to spend longer than reasonable time until the interviewee found the vaccination card, it is possible that we underestimated the proportion of card retention. Asking for detailed information about the vaccination card (e.g. whether the card was actually given or enquiring about the reasons why it was not available) may have been a way to overcome this limitation. We also conducted the surveys shortly after completion of vaccination activities so to increase the likelihood of card retention in the population. Secondly, the Phase I evaluation was done after mop-up activities so it was impossible to guide further vaccination activities in the district with the results of the evaluation. Thirdly, in Phase II, more females were surveyed than males, which may have biased the results if vaccination coverage is associated with gender. We were not able to check this association due to the small sample size in our survey. Lastly, if any adult member of the household or the individual selected for the survey were not reachable within a reasonable amount of time (e.g. was not spending the night away) before the completion of the cluster we instructed the surveyors to move to the subsequent household. Although this is a common practice, it may have biased the survey results in the sense that individuals present in the house at daytime had better chances of being selected in the survey [15].

In conclusion, our results suggest that vaccine coverage was satisfactory in Niamey and Tillabéri, although a number of unvaccinated individuals may exist, especially in the adult population and some districts. Combined use of LQAS and cluster sampling techniques allowed us to evaluate the campaigns at macro- (regional) and micro- (subpopulation and district) levels with greater timeliness and feasibility. These survey designs are complementary, cluster sampling being most useful to estimate coverage, LQAS or CLQAS to detect under-immunized pockets in certain populations or areas.

Ideally independent evaluations should be conducted at the end [26] or immediately after [25] the campaign to guide appropriate mop-up actions in close contact with local health authorities. Population estimates should be revised or updated in the country to improve the quality of the micro-planning and allocation of resources for future campaigns. In addition to enquiring about reasons for non-vaccination, we recommend including questions to investigate also the reasons for the absence of vaccination cards in order to plan appropriate measures to increase card retention in the population.
